# Pulmonary tumor thrombotic microangiopathy: A case report and literature review

**DOI:** 10.1097/MD.0000000000043284

**Published:** 2025-07-25

**Authors:** Lingling Yao, Jun Qin, Jianing Wang, Jinghua Liu

**Affiliations:** a Center for Coronary Artery Disease (CCAD), Beijing Anzhen Hospital, Capital Medical University, and Beijing Institute of Heart, Lung and Blood Vessel Diseases, Beijing, China; b Cardiac Care Unit (CCU), Renmin Hospital, Hubei University of Medicine, Shiyan, Hubei Province, China; c Department of Hematology, Renmin Hospital, Hubei University of Medicine, Shiyan, Hubei Province, China; d Department of Cardiology, Renmin Hospital, Hubei University of Medicine, Shiyan, Hubei Province, China.

**Keywords:** cancer, malignancy, pulmonary hypertension, pulmonary tumor thrombotic microangiopathy

## Abstract

**Rationale:**

Pulmonary tumor thrombotic microangiopathy (PTTM) is a rare malignancy-associated condition characterized by progressively worsening dyspnea, dry cough, hypoxemia, pulmonary hypertension, right-sided heart failure, and sudden death.

**Patient concerns:**

A 60-year-old female presented with intermittent dry cough, dyspnea, and chest pain.

**Diagnoses:**

The patient was suspected of having PTTM with a pancreatic primary malignancy, based on admission findings including percutaneous oxygen saturation of 88%, respiratory alkalosis on blood-gas analysis, and elevated levels of d-dimer, NT-proBNP, and multiple tumor markers, combined with computed tomography angiography results showing enlarged lymph nodes (mediastinum, bilateral hila, right cardiophrenic angle, retroperitoneum) and a blurred peripancreatic space.

**Interventions:**

Diagnostic investigations included physical examination, blood-gas analysis, laboratory tests (d-dimer, NT-proBNP, tumor markers), and computed tomography angiography of thoracic/abdominal vessels. (No therapeutic interventions detailed).

**Outcomes:**

The patient experienced 4 episodes of sudden clinical deterioration, suffered cardiac arrest, and died 9 days after admission. Autopsy was declined by the family.

**Lessons:**

PTTM must be considered in the differential diagnosis for patients presenting with dyspnea and new-onset severe pulmonary hypertension without other obvious etiology, particularly when malignancy is suspected.

## 1. Introduction

Pulmonary tumor thrombotic microangiopathy (PTTM) is a rare but fatal condition caused by metastatic cancer, pathologically characterized by nonocclusive small tumor emboli in pulmonary arterioles, diffuse thrombotic microvascular embolism, fibrous cell proliferation in pulmonary arterioles, luminal stenosis, and pulmonary vascular remodeling.^[1,2]^ An increasing frequency of PTTM has been reported, with a prevalence ranging from 1.3% to 16.7% in cancer patients.^[1,3,4]^ PTTM typically manifests as progressive dyspnea, dry cough, hypoxemia, and pulmonary hypertension without evidence of pulmonary embolism.^[5,6]^ Due to the rapid exacerbation of this condition, early diagnosis is crucial for reducing mortality. Here, we report a case of PTTM, initially presenting with a dry cough, to enhance clinicians’ understanding of this disease and to emphasize the importance of early diagnosis.

## 2. Case presentation

A 60-year-old female presenting with intermittent dry cough, chest distress, dyspnea, and chest pain was admitted to the People’s Hospital Affiliated to Hubei University of Medicine. One week prior to admission, the patient visited an outpatient clinic where bronchoscopy revealed acute bronchial inflammation. Pulmonary function tests showed a slight decrease in diffusion capacity and a negative reversibility test. Anti-inflammatory and antitussive treatments provided minimal relief, and her symptoms worsened, leading to her hospital admission. Her past medical history included hypertension, coronary heart disease, diabetes mellitus, cerebral hemorrhage, and varicose veins in the lower extremities, with no prior history of cancer. In addition, the patient underwent coronary stent implantation 1 year ago. On physical examination, her percutaneous oxygen saturation was 88%. Clinical findings included poor mental status, an acutely ill appearance, a semi-recumbent position, a few moist rales auscultated in both lower lungs, and mild bilateral lower limb edema.

Computed tomography angiography (CTA) covering the major thoracic, abdominal, and pulmonary vessels revealed no obvious abnormalities in these large vessels. However, enlarged lymph nodes were noted in the mediastinum, bilateral hila, right cardiophrenic angle, and retroperitoneum. The peripancreatic space appeared blurred, and a mixed-density nodule, suspected to be a teratoma, was observed in the left adnexal area. Color Doppler ultrasound of the bilateral lower extremities revealed atherosclerosis and a solid hypoechoic area in the right thigh’s superficial vein, suggestive of great saphenous vein thrombosis. Initial electrocardiogram, echocardiography, and chest ultrasonography showed no significant abnormalities.

Laboratory findings included respiratory alkalosis and elevated levels of N-terminal pro-brain natriuretic peptide (NT-proBNP), D-dimer, carcinoembryonic antigen, carbohydrate antigen (CA) 125, CA153, CA199, CA724, human chorionic gonadotropin, cytokeratin-19-fragment, and pro-gastrin-releasing peptide.

**Figure 1.**
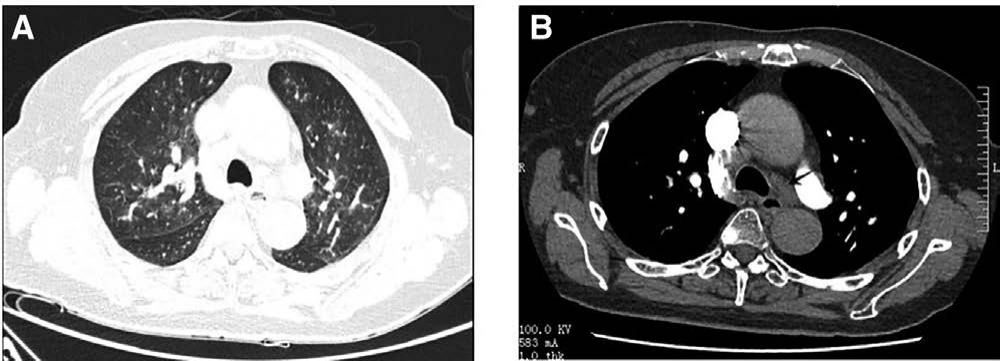
Chest computed tomography of the patient with pulmonary tumor microangiopathy as the disease aggravated. (A) The lung window showed that thickened lung texture and multiple patchy shadows. (B) The window of mediastinum revealed that multiple lymph nodes were enlarged.

**Figure 2.**
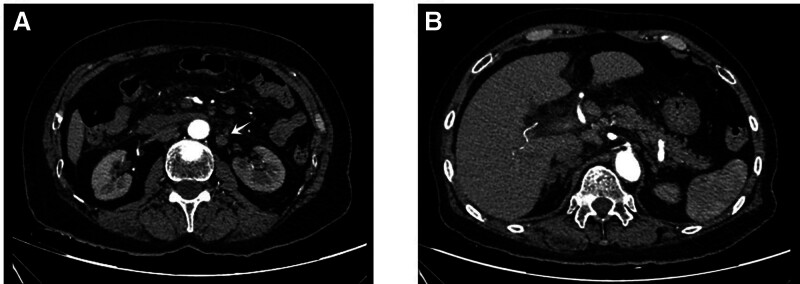
Enhanced-contrast computed tomography of the patient with pulmonary tumor microangiopathy as the disease aggravated. (A) Multiple lymph nodes were enlarged in upper abdomen and retroperitoneum. (B) The peripancreatic space was blurred.

**Figure 3.**
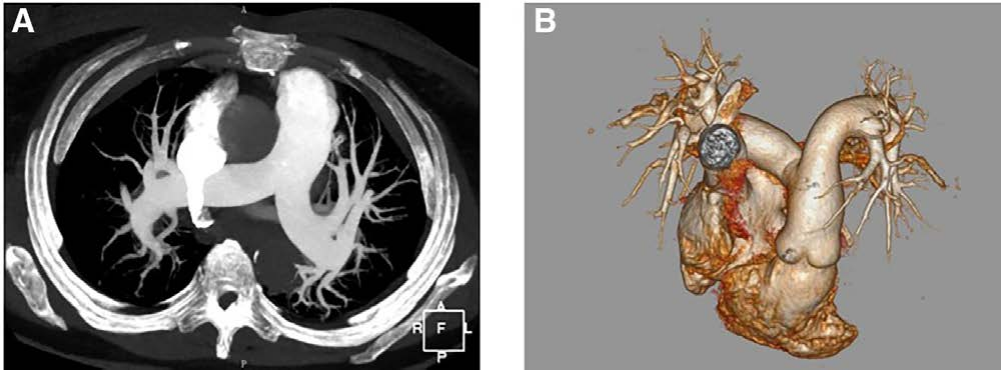
Pulmonary computed tomography angiography of the patient with pulmonary tumor thrombotic microangiopathy as the disease aggravated. (A) The diameter of the main pulmonary artery was widened (35 mm), suggesting pulmonary hypertension. (B) Three-dimensional reconstruction of pulmonary vessels indicated that there was no filling defect in the pulmonary artery.

Two days post-admission, the patient’s condition deteriorated, with worsening cough, mild hemoptysis, chest pain, dyspnea, tachycardia, and decreased oxygen saturation. Physical reexamination revealed bibasilar wet rales. A repeat echocardiogram at this time showed pulmonary hypertension, and her NT-proBNP levels continued to rise. Subsequent chest CT (Fig. 1) and contrast-enhanced whole-abdomen CT (Fig. 2) revealed bilateral pulmonary edema; other positive findings were similar to the initial CTA. An abdominal color Doppler ultrasound demonstrated fullness of the pancreatic head with blurring of the surrounding tissue planes. An anechoic area, possibly representing effusion, was noted on the right side of the pancreatic head, and multiple retroperitoneal lymph nodes were enlarged. Her symptoms transiently improved with diuretic therapy, bronchodilators, and noninvasive ventilation. However, her condition subsequently exacerbated, with further decreases in oxygen saturation, rising pulmonary pressure and NT-proBNP levels, and the development of type 1 respiratory failure. Consequently, a planned enhanced MRI was not performed due to her instability. Treatment included continued noninvasive ventilation, diuretics, a recombinant human brain natriuretic peptide infusion, antiasthmatic medications, and mucolytics. Following a period of symptom relief, a repeat pulmonary CTA was performed, which again showed no evidence of pulmonary embolism (Fig. 3). However, the main pulmonary artery diameter was dilated to 35 mm, indicative of pulmonary hypertension. Additionally, her NT-proBNP levels remained persistently elevated. Her condition acutely worsened again 9 days after admission, culminating in cardiac arrest. Despite comprehensive resuscitation efforts, the patient died. Her family declined an autopsy.

## 3. Discussion

The patient initially presented with a dry cough, which rapidly progressed to dyspnea, chest pain, hemoptysis, and syncope. D-dimer and blood lactate levels were significantly elevated, and her hypoxemia worsened. Serial echocardiograms demonstrated a progressive increase in pulmonary artery pressure, while acute pulmonary embolism was excluded by 2 separate pulmonary CTA examinations. Significant elevations in multiple tumor markers (notably CA199, carcinoembryonic antigen, and CA724), along with ultrasound and CT findings of a blurred peripancreatic space and multiple enlarged lymph nodes, strongly suggested an underlying pancreatic malignancy. Due to her poor clinical condition and the rapid progression of her illness, enhanced MRI and PET-CT examinations were not performed, and her family declined a lung needle biopsy. The patient died 9 days after admission, and as her family declined an autopsy, no definitive pathological evidence was obtained. However, based on her clinical characteristics and the exclusion of other potential causes of pulmonary hypertension, a presumptive diagnosis of PTTM was made.

PTTM was first reported by Von Herbay.^[1]^ The pathophysiology of PTTM is not completely understood. However, it is characterized by the presence of tumor cells, fibrin deposition, and fibroblastic intimal hyperplasia within the pulmonary capillary lumen, leading to pulmonary hypertension and right-sided heart failure.^[7,8]^ This pathophysiological process is thought to be mediated by the release of various growth factors and vasoactive substances. Gastric cancer, particularly adenocarcinoma, is the most common primary malignancy associated with PTTM.^[9]^ PTTM has also been reported in other tumors, including breast cancer, lung cancer, prostate cancer, cervical cancer, colon cancer, hepatocellular carcinoma, and pancreatic cancer.^[10,11]^ A common feature is the presence of distant metastases, frequently involving lymph nodes. PTTM is predominantly found in patients with advanced cancer, although in some cases, PTTM may be diagnosed before the primary tumor is identified.^[12]^ Early diagnosis is exceedingly difficult, and many cases are only confirmed by postmortem autopsy. CT-guided or transbronchial lung biopsy remains the gold standard for antemortem diagnosis.^[13]^ However, patients often suffer from progressive cardiopulmonary failure, rendering them too unstable for invasive procedures. Consequently, obtaining tissue specimens can be challenging, and in China, most cases are diagnosed clinically without pathological confirmation.

The clinical symptoms of PTTM are nonspecific, with most patients manifesting progressive dyspnea, cough, and hypoxemia.^[14]^ Clinical signs are usually associated with pulmonary hypertension and right heart dysfunction. Specific chest radiograph findings are variable and often nondiagnostic. Chest CT in PTTM patients may reveal ground-glass opacities, small nodules, bronchovascular bundle thickening, lung consolidation, and enlarged mediastinal and/or hilar lymph nodes.^[15]^ Pulmonary CTA may show signs of pulmonary hypertension such as widening of the main pulmonary artery and enlargement of the right heart, but there is no evidence of pulmonary embolism. The nonspecific clinical and radiological features of PTTM make early diagnosis challenging for clinicians. The prognosis for patients with PTTM is extremely poor, with a reported median survival of only 9 days from the onset of oxygen dependency.^[4]^ Increased clinical vigilance for PTTM is crucial for facilitating early diagnosis and intervention. Therefore, PTTM should be considered in the differential diagnosis of patients presenting with unexplained dyspnea, hypoxemia, new-onset pulmonary hypertension, and significantly elevated D-dimer levels, particularly in the absence of pulmonary embolism, regardless of whether a tumor has been definitively identified. Evaluation of tumor markers and PET/CT imaging can aid in identifying the primary lesion. If feasible, biopsy of the suspected primary lesion or lung tissue, or right heart catheterization with pulmonary microvascular cytology, could be considered based on the patient’s clinical status.

In clinical practice, PTTM should also be distinguished from pulmonary embolism, chronic thromboembolic pulmonary hypertension (CTEPH), pulmonary veno-occlusive disease (PVOD), and pulmonary lymphangitic carcinomatosis. Pulmonary CTA in cases of pulmonary embolism may show pulmonary artery filling defects, wedge-shaped high-density shadows, linear high-density shadows, or disc-like atelectasis in the lung fields.^[16]^ This patient had an acute clinical course, no prior history of pulmonary embolism, and 2 negative pulmonary CTA examinations, thereby excluding acute pulmonary embolism. CTEPH, a rare complication of acute pulmonary embolism, can present with symptoms ranging from exertional dyspnea to overt right heart failure.^[17]^ However, cough is a common symptom in PTTM but less common in CTEPH. CTEPH typically progresses over a prolonged period, whereas PTTM has a rapid course. Chest CT in CTEPH often reveals diffuse mosaic attenuation, wedge-shaped opacities, and prominent bronchial artery circulation, features typically absent in PTTM. PVOD is primarily characterized by lesions in the pulmonary venous system, where venules undergo thrombosis and become occluded by fibrous tissue, leading to luminal stenosis or obstruction.^[18]^ The clinical course of PVOD is generally protracted. In contrast, this patient’s echocardiographic findings rapidly progressed from normal to overt pulmonary hypertension within days, inconsistent with PVOD. Chest imaging in pulmonary lymphangitic carcinomatosis typically shows multiple bilateral lung nodules, thickened interlobular septa, and beaded changes, findings not consistent with this patient’s CT.^[19]^ The pre-admission bronchoscopy revealed only acute inflammatory changes, further making pulmonary lymphangitic carcinomatosis less likely. Based on the 2022 ESC/ERS Guidelines for the diagnosis and treatment of pulmonary hypertension,^[20]^ a diagnosis of PTTM was supported by the elevated levels of multiple tumor markers and characteristic radiological findings in the context of unexplained severe pulmonary hypertension.

Currently, no randomized trials have evaluated specific drug efficacy for pulmonary hypertension associated with tumor embolism. Case reports suggest that chemotherapy or targeted therapy directed at the underlying malignancy should be initiated promptly upon diagnosis of PTTM.^[21]^ Other treatment regimens that improve pulmonary pressures and/or cardiac output may also prolong survival. Imatinib, a platelet-derived growth factor receptor inhibitor, has potential for reverse remodeling due to its anti-proliferative, pro-apoptotic, and vasoconstrictive-modulating effects.^[22]^ One study found that imatinib could reduce pulmonary pressure and improve survival in this context.^[23]^ More recently, anlotinib was shown to improve pulmonary hypertension and respiratory function in a PTTM patient with gastric cancer.^[24]^ Patients with PTTM are at high risk for venous thromboembolism, and anticoagulant or antiplatelet therapy may offer benefits. Patients may also benefit from anti-inflammatory treatments and targeted therapies aimed at reducing pulmonary pressure with vasoactive substances.^[25,26]^ Additionally, veno-arterial extracorporeal membrane oxygenation can provide effective rescue therapy for patients experiencing rapid deterioration and hemodynamic instability.^[27]^ To improve outcomes and reduce mortality, comprehensive treatment strategies are essential.

It is crucial to acknowledge the limitations inherent in the diagnostic process of this case. The most significant limitation is the absence of definitive pathological confirmation, which remains the gold standard for diagnosing PTTM. This absence was a direct consequence of the patient’s rapid and fulminant clinical course, which precluded invasive diagnostic procedures such as CT-guided or transbronchial lung biopsy due to her critical and deteriorating condition. Furthermore, the family’s understandable declination of an autopsy following the patient’s death prevented postmortem histological examination. Consequently, the diagnosis of PTTM, although strongly supported by the constellation of clinical, laboratory, and radiological findings, remains presumptive. Additionally, the suspected primary tumor site (pancreatic malignancy) was not histologically confirmed due to these same constraints: patient instability, rapid decline, and family refusal of biopsy. Enhanced MRI and PET-CT, which could have provided further evidence of malignancy and potential metastatic burden, were also not feasible during the patient’s short and unstable hospital course. Despite these limitations, this case vividly illustrates the characteristic rapid progression, diagnostic challenges, and often catastrophic outcome associated with PTTM. It underscores the critical importance of maintaining a high index of clinical suspicion based on the presenting features (such as unexplained rapid-onset severe pulmonary hypertension, hypoxemia, elevated D-dimer without pulmonary embolism, and evidence of occult malignancy) particularly when pathological proof is unattainable, as is often the case in this devastating condition.

## 4. Conclusion

PTTM must be considered as a differential diagnosis in patients presenting with dyspnea and new-onset severe pulmonary hypertension without any other obvious etiology, especially if an underlying malignancy is suspected. If PTTM is suspected, identifying the primary cancer site is crucial, and prompt initiation of comprehensive treatment, often including chemotherapy directed at the underlying malignancy, is necessary.

## Author contributions

**Conceptualization:** Lingling Yao.

**Data curation:** Lingling Yao.

**Formal analysis:** Lingling Yao, Jun Qin.

**Investigation:** Jun Qin.

**Methodology:** Jianing Wang.

**Project administration:** Jianing Wang, Jinghua Liu.

**Supervision:** Jinghua Liu.

**Writing – original draft:** Lingling Yao.

**Writing – review & editing:** Jinghua Liu.

## References

[1] von Herbay A Illes A Waldherr R Otto HF. Pulmonary tumor thrombotic microangiopathy with pulmonary hypertension. Cancer. 1990;66:587–92. 2163747 10.1002/1097-0142(19900801)66:3<587::aid-cncr2820660330>3.0.co;2-j

[2] Ramakrishnan P Dahiya G Lindstrom M Thenappan T. Pulmonary tumor thrombotic microangiopathy: exploration into current diagnostic aids and therapeutics. Pulm Circ. 2023;13:e12278. 37593090 10.1002/pul2.12278 PMC10427771

[3] Chinen K Tokuda Y Fujiwara M Fujioka Y. Pulmonary tumor thrombotic microangiopathy in patients with gastric carcinoma: an analysis of 6 autopsy cases and review of the literature. Pathol Res Pract. 2010;206:682–9. 20554399 10.1016/j.prp.2010.05.002

[4] Uruga H Fujii T Kurosaki A. Pulmonary tumor thrombotic microangiopathy: a clinical analysis of 30 autopsy cases. Intern Med. 2013;52:1317–23. 23774540 10.2169/internalmedicine.52.9472

[5] Kim KA Jung MH. Pulmonary tumor thrombotic microangiopathy: an under-recognized potentially fatal cause of pulmonary hypertension. Korean Circ J. 2023;53:185–8. 36914607 10.4070/kcj.2023.0019 PMC10011223

[6] Schwartz BP Tracy P Hon S Farber HW Udelson JE. Pulmonary tumor thrombotic microangiopathy as a cause of pulmonary hypertension. JACC Case Rep. 2021;3:1029–31. 34317678 10.1016/j.jaccas.2021.04.013 PMC8311364

[7] Fujita J Hummel K Xu Y. Pulmonary tumor thrombotic microangiopathy caused by metastatic ovarian high-grade serous carcinoma: a case report and literature review. Cardiovasc Pathol. 2023;65:107526. 36781067 10.1016/j.carpath.2023.107526

[8] Tomioka T Tanaka S Takeuchi H. Pulmonary tumor thrombotic microangiopathy with administration of pulmonary vasodilator resulting in clinical improvement prior to final diagnosis. Am J Case Rep. 2021;22:e933867. 34611123 10.12659/AJCR.933867 PMC8503794

[9] Choudhury S Mishra SB Panda S. Pulmonary tumor thrombotic microangiopathy in a patient of gastric carcinoma: a rare entity. Indian J Crit Care Med. 2022;26:763–4. 35836618 10.5005/jp-journals-10071-24230 PMC9237147

[10] Morita S Kamimura K Abe H. Pulmonary tumor thrombotic microangiopathy of hepatocellular carcinoma: a case report and review of literature. World J Gastroenterol. 2019;25:6949–58. 31908398 10.3748/wjg.v25.i48.6949 PMC6938728

[11] Rudolf F Baschong A Bilecen D Aceto N Vetter M. Pulmonary tumor thrombotic microangiopathy in a patient with rapid progressive triple-negative breast cancer. Case Rep Oncol. 2024;17:277–82. 38371170 10.1159/000535873 PMC10870124

[12] Amonkar GP Jashnani KD Pallewad S. Pulmonary tumor thrombotic microangiopathy in an unknown primary cancer. Lung India. 2014;31:410–2. 25378856 10.4103/0970-2113.142153 PMC4220330

[13] Onoda H Imamura T Inao K Kinugawa K. How to diagnose and treat pulmonary tumor thrombotic microangiopathy. Int Heart J. 2020;61:409–12. 32173712 10.1536/ihj.19-549

[14] Noiri JI Taniguchi Y Izawa Y. Pulmonary tumor thrombotic microangiopathy due to early gastric carcinoma in a patient with no antemortem findings suggestive of primary malignancy. Pulm Circ. 2024;14:e12359. 38550874 10.1002/pul2.12359 PMC10973772

[15] Yamada D Murakami M Matsusako M Tamura T Kurihara Y. Pulmonary tumor thrombotic microangiopathy appearance on dual-energy computed tomography. Am J Respir Crit Care Med. 2021;203:759–60. 33197218 10.1164/rccm.202004-0907IM

[16] Trott T Bowman J. Diagnosis and management of pulmonary embolism. Emerg Med Clin North Am. 2022;40:565–81. 35953217 10.1016/j.emc.2022.05.008

[17] Valerio L Mavromanoli AC Barco S. Chronic thromboembolic pulmonary hypertension and impairment after pulmonary embolism: the FOCUS study. Eur Heart J. 2022;43:3387–98. 35484821 10.1093/eurheartj/ehac206 PMC9492241

[18] Solinas S Boucly A Beurnier A. Diagnosis and management of pulmonary veno-occlusive disease. Expert Rev Respir Med. 2023;17:635–49. 37578057 10.1080/17476348.2023.2247989

[19] Jreige M Dunet V Letovanec I. Pulmonary lymphangitic carcinomatosis: diagnostic performance of high-resolution CT and (18)F-FDG PET/CT in correlation with clinical pathologic outcome. J Nucl Med. 2020;61:26–32. 31227574 10.2967/jnumed.119.229575

[20] Humbert M Kovacs G Hoeper MM. 2022 ESC/ERS Guidelines for the diagnosis and treatment of pulmonary hypertension. Eur Respir J. 2023;61:2200879. 36028254 10.1183/13993003.00879-2022

[21] Kamidani R Kumada K Okada H. Postmortem diagnosis of pulmonary tumor thrombotic microangiopathy with rapid exacerbation in a patient with gastric cancer: a case report. Int J Emerg Med. 2021;14:53. 34525938 10.1186/s12245-021-00377-2 PMC8444540

[22] Xin Y Cereda M Yehya N. Imatinib alleviates lung injury and prolongs survival in ventilated rats. Am J Physiol Lung Cell Mol Physiol. 2022;322:L866–72. 35438574 10.1152/ajplung.00006.2022 PMC9142156

[23] Kubota K Shinozaki T Imai Y Kario K. Imatinib dramatically alleviates pulmonary tumour thrombotic microangiopathy induced by gastric cancer. BMJ Case Rep. 2017;2017:bcr2017221032 . 10.1136/bcr-2017-221032 PMC5589033 28882938

[24] Wang Y Ning WW Jin YF. Anlotinib dramatically improved pulmonary hypertension and hypoxia caused by Pulmonary Tumor Thrombotic Microangiopathy (PTTM) associated with gastric carcinoma: a case report. Thromb J. 2023;21:33. 36973680 10.1186/s12959-023-00477-4 PMC10041787

[25] Godbole RH Saggar R Kamangar N. Pulmonary tumor thrombotic microangiopathy: a systematic review. Pulm Circ. 2019;9:1–13. 10.1177/2045894019851000 PMC6540517 31032740

[26] Tran LK Gross LM Hagley P Minkin R. Pulmonary hypertension in metastatic breast cancer: a case of pulmonary tumour thrombotic microangiopathy. BMJ Case Rep. 2019;12:e229715. 10.1136/bcr-2019-229715 PMC6731773 31488441

[27] Iwashita Y Hiramoto T Suzuki K Hashizume R Maruyama K Imai H. Possibility of venoarterial extracorporeal membranous oxygenator being a bridging therapy for hemodynamic deterioration of pulmonary tumor thrombotic microangiopathy prior to initiating chemotherapy: a case report. Medicine (Baltimore). 2018;97:e12169. 30212945 10.1097/MD.0000000000012169 PMC6155969

